# Development of face recognition: Dynamic causal modelling of MEG data

**DOI:** 10.1016/j.dcn.2017.11.010

**Published:** 2017-11-23

**Authors:** Wei He, Blake W. Johnson

**Affiliations:** a Department of Cognitive Science, Macquarie University, New South Wales 2109, Australia; b Australian Research Council Centre of Excellence in Cognition and Its Disorders, Macquarie University, New South Wales 2109, Australia

**Keywords:** MEG, Face recognition, Repetition, DCM, M250, M170

## Abstract

Electrophysiological studies of adults indicate that brain activity is enhanced during viewing of repeated faces, at a latency of about 250 ms after the onset of the face (M250/N250). The present study aimed to determine if this effect was also present in preschool-aged children, whose brain activity was measured in a custom-sized pediatric MEG system. The results showed that, unlike adults, face repetition did not show any significant modulation of M250 amplitude in children; however children’s M250 latencies were significantly faster for repeated than non-repeated faces. Dynamic causal modelling (DCM) of the M250 in both age groups tested the effects of face repetition within the core face network including the occipital face area (OFA), the fusiform face area (FFA), and the superior temporal sulcus (STS). DCM revealed that repetition of identical faces altered both forward and backward connections in children and adults; however the modulations involved inputs to both FFA and OFA in adults but only to OFA in children. These findings suggest that the amplitude-insensitivity of the immature M250 may be due to a weaker connection between the FFA and lower visual areas.

## Highlights

• M250 shows amplitude sensitivity to face repetition in adults, but latency sensitivity in children.
• Earlier peaks (M100 and M170) show no sensitivity to face repetition in either group.
• Face repetition modulates reciprocal connections between OFA and FFA in both groups.
• Repetition modulates low-level visual projections to OFA and FFA in adults but only to OFA in children.

## 1 Introduction

The human face conveys an extremely rich set of information concerning identity, gender, emotion, and other important social cues. Interpretation of this wealth of information is essential for social interactions and requires elaborative processes in multiple brain regions, with a bias toward right hemisphere structures (Haxby et al., 2000, Rossion, 2014). Functional magnetic resonance imaging (fMRI) studies in healthy adults show a stronger blood-oxygen-level dependent (BOLD) signal in the lateral inferior occipital gyrus (i.e., the occipital face area, OFA; Gauthier et al., 2000a, Rossion et al., 2003) and the middle fusiform gyrus (i.e., fusiform face area, FFA; Kanwisher and Yovel, 2006) when processing faces or invariant facial aspects (e.g., eyes) compared to other object categories (e.g., cars). Moreover, evidence from fMRI-neural adaptation or repetition suppression studies demonstrates that BOLD activities in both the OFA and FFA are increased for a sequence of unique faces compared to repeated faces (Eger et al., 2004, Ewbank et al., 2012, Gauthier et al., 2000b, Schiltz and Rossion, 2006). Such *“*release from adaptation*”* suggests that similar populations of face-selective neurons may function in both categorizing (e.g., ‘it is a face, not a car’) and identifying faces (e.g., ‘it is face A, not face B’).

Electrophysiological recordings using magneto-/electro-encephalography (M/EEG) have provided information about the temporal sequencing of face-specific processing stages (Olivares et al., 2015, Rossion, 2014). Two important M/EEG components, the M/N170 and the M/N250, are believed to index structural (∼130–200 ms; e.g., Bentin et al., 1996, Rossion and Jacques, 2008) and identity (∼220–300 ms; e.g., Itier et al., 2006, Schweinberger et al., 2007, Schweinberger et al., 2002, Walther et al., 2013) encoding stages of facial attributes. A recent MEG study in young adults reported that the right fusiform gyrus exhibits an early reduction in its activity at around 150 ms for category-level repetitions of face stimuli (e.g., face-to-different-face) and a late reduction between 200 and 300 ms for item-level repetitions (face-to-same-face) (Simpson et al., 2015). Taken together, the fMRI and MEG data suggest that a common population of neurons in the OFA and FFA carries out categorization and identification of faces in two sequential stages.

Given the social importance of face perception, one might assume the underlying brain regions should mature quite early. In fact, this issue is still strongly debated in the literature. Compelling support for early maturation has been reported by recent fMRI and M/EEG studies, showing that the activation and response patterns of the FFA (Cantlon et al., 2010, Haist et al., 2013) and the M/N170 (He et al., 2015, Kuefner et al., 2010) are stable from age 4–5 years (McKone et al., 2012). On the other hand, there is a wealth of fMRI data showing that the brain regions comprising the face network continue to mature to adolescence and beyond ([Bibr bib0030][Bibr bib0145], Song et al., 2015).

In adults, the repetition of unfamiliar faces is known to modulate the amplitude of M250, indicating the initial encoding of individual face exemplars (Schweinberger, 2011, Schweinberger et al., 2004, Schweinberger et al., 2002, Walther et al., 2013). It remains unclear whether there is a comparable effect in children’s brain responses. One EEG study on 7-month-old infants reported a more negative N290 (a precursor of the adult N170) amplitude for novel female faces compared to 1-back repeated female faces (with one intervening different face) (Righi et al., 2014). However, a later infant EEG study using human faces, ape faces, and houses in an immediate repetition paradigm reported that, unlike the adult response, the N290 ampliutde was modulated at the level of the basic categorization (human, ape, or house) but not the individual-level representation (Righi et al., 2014, Peykarjou et al., 2014). Another recent EEG study found no N290 amplitude effect, but did find a reduced N290 latency for repeated faces, which the authors interpreted to suggest faster processing of the repeated faces (Peykarjou et al., 2016).

In older children, two recent M/EEG studies using face repetition tasks have reported a frontal negative component between 250 and 600 ms in children as young as 8-years-old that is sensitive to face repetitions, with a larger amplitude for immediately repeated faces than novel/non-repeated faces (Itier and Taylor, 2004). Source reconstruction on a similar component in 6–7 year-olds showed enhanced activation in the right hippocampus to repeated (but unfamiliar) faces (Taylor et al., 2011b). It remains unclear how this frontally-distributed component may be related to the occipitotemporal M/N250 component reported in adults.

While there have been a few electrophysiological and neuroimaging studies of face repetition effects in infancy, and in school-aged children, there have been no studies of children of intermediate ages. There are two main reasons for the lack of neuroimaging studies on face recognition in early childhood. Firstly, preschool children have a limited capacity for the sustained attentive vigilance and behaviour control typically required in such experiments (Brown and Jernigan, 2012). Secondly, most neuroimaging systems with adult-sized head coils (fMRI) and helmet dewars (MEG) are poorly optimised for use with the smaller heads of children (Johnson et al., 2010). The advantage of using a custom-sized pediatric MEG system with pre-school aged children has been demonstrated in our previous work showing a robust face-sensitive M170 response in a group of 3- to 6-year-old children (He et al., 2014a, He et al., 2014b, He et al., 2015); a response that has not been detected in previous studies using a conventional adult MEG system (Kylliainen et al., 2006, Taylor et al., 2010). Furthermore, using dynamic causal modelling (DCM), we were able to elucidate developmental changes in the connectivity of the core face network comprised of the OFA, FFA, and superior temporal sulcus (STS) (He et al., 2015).

In the present study, we aimed to extend our previous pediatric MEG work on the N170 to the subsequent M250 stage of face processing in healthy preschool aged children. To this end, we used a passive viewing repetition paradigm with an orthogonal visual detection task (Schweinberger et al., 2007). Repetitions were presented in a 0-lag and passive viewing design to minimise the cognitive and attentional demands on the children. We examined whether an M250 effect is detectable in children (using a pediatric MEG system); and compared the effective connectivity of the OFA and FFA underlying the neural responses obtained in both groups.

## 2 Material and methods

### 2.1 Participants

Data were collected from 10 typically-developing children (4 M, aged 5.3 ± 0.83 years, range 4–6 years) and 11 healthy adults (7 M, aged 24 ± 5.76 years, range 18–33 years). All participants were right-handed with normal or corrected to normal vision. Data from an additional 12 participants (10 children and 2 adults) were excluded due to non-compliance (6 children), excessive head movement (>10 mm throughout the whole session causing loss of more than 40% of trials, 4 children), and technical problems during data acquisition (2 adults). The experimental procedures were approved by the Human Participants Ethics Committee at Macquarie University. Written informed consent was obtained from the adult participants and from the parents/guardians of the children prior to the experiment.

### 2.2 Experimental procedure

Upon arriving at the laboratory, participants were familiarized with the magnetically shielded room (MSR) where they were tested in a supine position with visual images projected onto a screen by video projectors situated outside the MSR room (child MEG projector: InFocus Model IN5108, Portland; Adult MEG projector: Sharp Notevision Model PG10S, Japan). Prior to MEG measurements, five head position indicators (HPI) were attached to a tightly fitting elastic cap. The 3D locations of the HPIs, fiducial landmarks and the shape of each participant's head were measured with a pen digitizer (Polhemus Fastrack, Colchester, VT). Then, children were tested using the child custom-sized 64-channel whole-head axial gradiometer MEG system (Model PQ1064R-N2m, KIT, Kanazawa, Japan), and adults were tested using the 160-channel whole-head axial gradiometer MEG system (Model PQ1160RN2, KIT, Kanazawa, Japan). The gradiometers of both systems have a 50 mm baseline and a 15.5 mm diameter positioned in a glass fibre reinforced plastic cryostat for measurement of the normal component of the magnetic field from the human brain (Kado et al., 1999). In both systems, neighbouring channels are 38 mm apart, and 20 mm from the outer dewar surface. The size of the dewar helmet of the child system was 53.4 cm. This was designed to fit 90% of heads of 5-year olds (for more details please refer to Johnson et al., 2010).

Both systems were situated within the same MSR and therefore environmental noise was equivalent. Stimuli consisted of 84 colour pictures, including 43 unfamiliar faces (24 male) and 41 cartoon alien pictures. Faces were posed with neutral expression and without glasses, earrings, facial hair or make-up. All pictures were trimmed to remove any background, including clothing and hair. Four blocks of 63 pictures were presented with 86 trials of immediate repetitions (0-lag) and 86 trials of no repetitions. No individual face appeared more than 3 times within a block. To ensure that participants maintained vigilance, they were required to press a button for catch trials of cartoon aliens, randomly embedded into the image stream. Brain responses to the catch trials and to faces presented immediately before or after the catch trials were not analysed further. Repeated, non-repeated, and catch trials were presented in a pseudo-randomized order. Even though each face was repeated only once after a varying number of intervening stimuli, participants might develop expectations about the nature of the next stimulus. To examine this possibility, we compared responses for the first and last blocks of trials and found no significant differences (see section 1 in the Supplementary Materials).

**Fig. 1:**
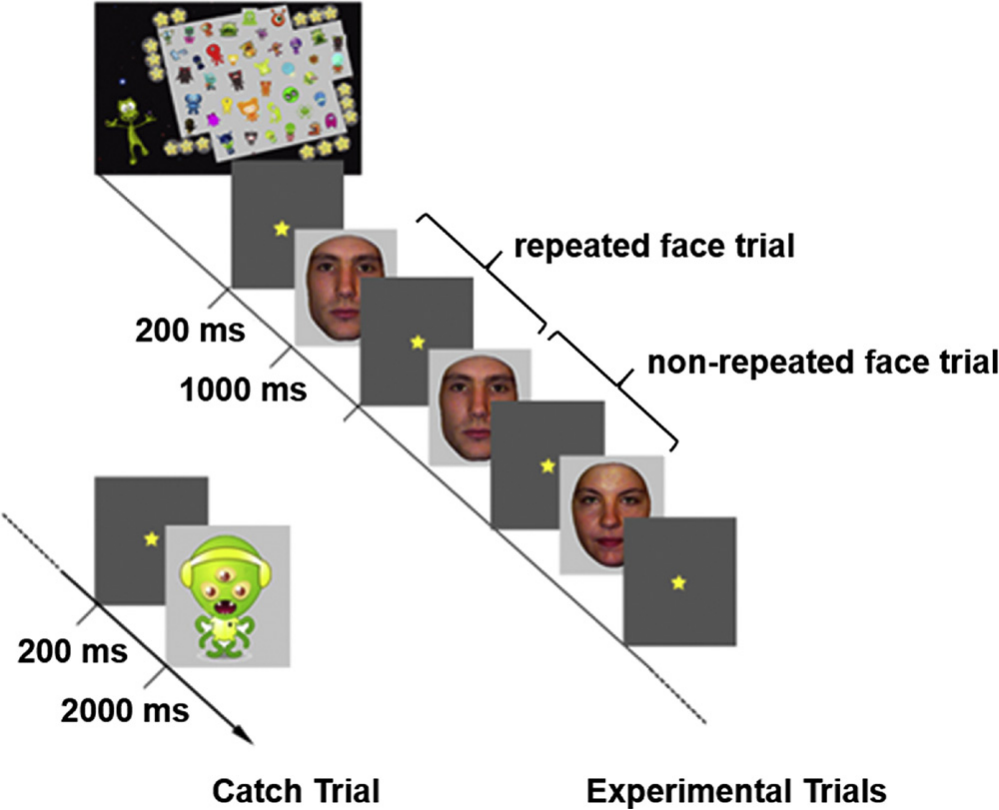
Schematic illustration of the child-friendly experimental paradigm. Faces were presented at a rate of 1000 ms, a fixation (star) interleaved between faces for 200 ms, and inter-stimulus interval was 1000 ± 50 ms. Each face was paired either with the same face (*repeated face trial*) or with a different face (*non-repeated face trial*), i.e., used twice in both 86 repeated or 86 non-repeated trials.

The experiment was programmed using Experiment Builder software (SR Research Ltd., Mississauga, Ontario, Canada). All pictures were presented within a light grey frame fitted into a rectangular area that subtended a visual angle of 3.10° × 4.58° in the adult system and 2.64° × 3.90° in the child system. The monocular gaze of the participant's right eye was monitored by an SR Research Eyelink 1000 eye-tracking system with a sampling rate of 1000 Hz (http://www.sr-research.com/EL_1000.html). Each trial began with a fixation point that appeared at the centre of the screen for 200 ms. Each stimulus was then presented for 1000 ms with the condition that eye fixations were maintained in the proximity of the fixation point. The mean inter-stimulus interval was 1000 ms (with a random jitter of 50 ms). Catch trials remained on the screen until a response was made or a maximum duration of 2000 ms occurred (Fig. 1). In both groups, participants responded to catch trials with accuracy greater than 98%.

### 2.3 MEG data acquisition

MEG data were recorded continuously using a sampling rate of 1000 Hz and an on-line bandpass of 0.03–200 Hz. Head locations were measured before and after each block; movement tolerance was a maximum of 5 mm in adults and 10 mm in children.

Off-line data processing was done using SPM8 (Litvak et al., 2011) and Fieldtrip (Oostenveld et al., 2011) toolboxes in MATLAB2013a (http://au.mathworks.com/products/matlab/). Data were low-pass filtered at 30 Hz, down-sampled to 250 Hz, high-pass filtered at 1.6 Hz, epoched around the time of stimulus onset (-100 to 600 ms), and baseline corrected. A baseline epoch was created at −800 to −100 ms from stimulus onset and concatenated to the main epoched data. Trials containing large amplitude artifacts were removed using the Fieldtrip visual artefact rejection method (z-score < 2). There were 86 trials for repeated face and non-repeated face (i.e., the first representation of the face) conditions respectively in each individual data, and approximately 98% of trials in each adult and 97% of trials in each child data survived the rejection procedure. Data from each participant was then co-registered with the individual dewar head location. For children who took breaks in-between acquisition blocks, data for each recording block were first co-registered with initial dewar head location files of individual blocks, and then transformed into a common sensor space averaged across all acquisition blocks using the realignment method implemented in Fieldtrip (Knosche, 2002). The same realignment of sensor space onto a common sensor space procedure was applied to individual data before group statistics were performed. Robust averaging was applied to average across trials within the two conditions, i.e., the repeated faces and non-repeated faces, for each sensor and participant (Litvak et al., 2011). Outliers were down-weighted by this method using an iterative robust general linear model (Wager et al., 2005), which calculates and reassigns weights to each sample of each trial according to how far it is from the mean response. To remove any high-frequency noise introduced by the robust averaging step, the averaged epoched data were low-pass filtered again at 30 Hz.

### 2.4 Sensor space analysis

To determine differences in the evoked magnetic brain responses between conditions at the sensor- level, we used topological inference (Chumbley and Friston, 2009), a method based on the random field theory and implemented in SPM8, to search and compare event-related fields (ERFs) in the entire sensor space. This method stringently corrects for multiple statistical comparisons by calculating the family-wise error (FWE) rate across 64 × 64 pixel image for each of the time points from 100 ms before to 600 ms after stimulus onset. In order to increase statistical power, we applied a sensor of interest (SOI) approach to select sensors where the ERFs to face stimuli were greater than baseline (FWE corrected *p <* 0.05). The time window for which the ERFs were greater than baseline within the SOI was identified as the relative time period of interest. This SOI procedure is orthogonal to the effect of interest because it averaged over repeated and non-repeated face stimuli. Subsequently, the mean amplitude for each sensor was then averaged across the SOI regions to produce a value for each subject and each condition to test for condition-specific effects within groups. We selected to calculate root-mean-square (RMS) waveforms as the sensor-level technique since it captures simultaneous activity across SOI clusters, is less susceptible to variations in head-sensor-positions, and is more comparable across different types of MEG gradiometers (Ozaki et al., 2012). Due to the fact that adults and children were measured with two different MEG systems, between-group comparison was inappropriate at the sensor level, and therefore only within-group comparisons were made to characterise the repetition effect in both groups. A two-sided paired *t*-test was performed on the averaged responses using IBM © SPSS © Statistics (v21.0). *P*-values for all pairwise multiple comparisons were corrected by false discovery rate (FDR, *q <* 0.05) (Benjamini and Hochberg, 1995).

### 2.5 Dynamic causal modelling (DCM) and bayesian model selection (BMS)

DCM for EEG/MEG uses generative neural mass models of the brain to characterise dynamic changes induced by experimental contextual modulations among brain regions, and therefore facilitates hypothesis testing in terms of making inferences about the direction and strength of effective connectivity within the neural architecture underlying the electromagnetic signals observed at the scalp level (David et al., 2006). All DCM analyses were performed using DCM10 as implemented in SPM8.

A canonical cortical mesh derived from the MNI template T1 image was co-registered and warped, in a non-linear manner, to match each participant’s digitised head-shape data. A single sphere model was used to compute leadfield used by the source reconstruction scheme implanted in the DCM inversion procedure (Litvak et al., 2011). Source regions defined here are estimated as priors (dipole locations or moments), from which Bayesian inversion of the DCM implicitly estimates the conditional density of the locations (16 mm^2^ Gaussian dispersion) and orientations (under uninformative or flat priors) (David et al., 2006, Kiebel et al., 2006).

**Fig. 2:**
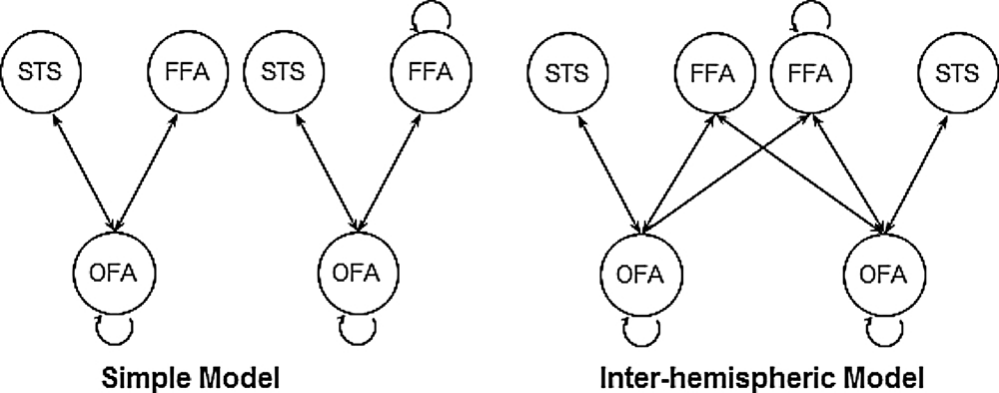
Model structures adapted from a previous developmental DCM-MEG study (He et al., 2015). The simple model structure contains connections between OFA, FFA and STS. The inter-hemispheric model includes extra inter-hemispheric connections from OFA to contralateral FFA. OFA, occipital face area (inferior occipital gyrus); FFA, fusiform face area (fusiform gyrus); STS, superior temporal sulcus.

DCM model space, consisting of the bilateral OFA (MNI coordinates: right: [42−77 −11]; left [-39, −81, −15]), FFA (right: [42−45 −27]; left [-39, −51, −24]), and the superior temporal sulcus (STS; right: [48−42 12]; left [-48, −42, 12]), was adapted from a previous developmental DCM-MEG study (He et al., 2015). The two model structures, as outlined in Fig. 2, are both comprised of reciprocal connections between OFA, FFA and STS within each hemisphere: the inter-hemispheric model space (the child winning model in He et al., 2015) includes extra inter-hemispheric connections from OFA to contralateral FFA on top of the simple model space (the adult winning model in He et al., 2015).

**Fig. 3:**
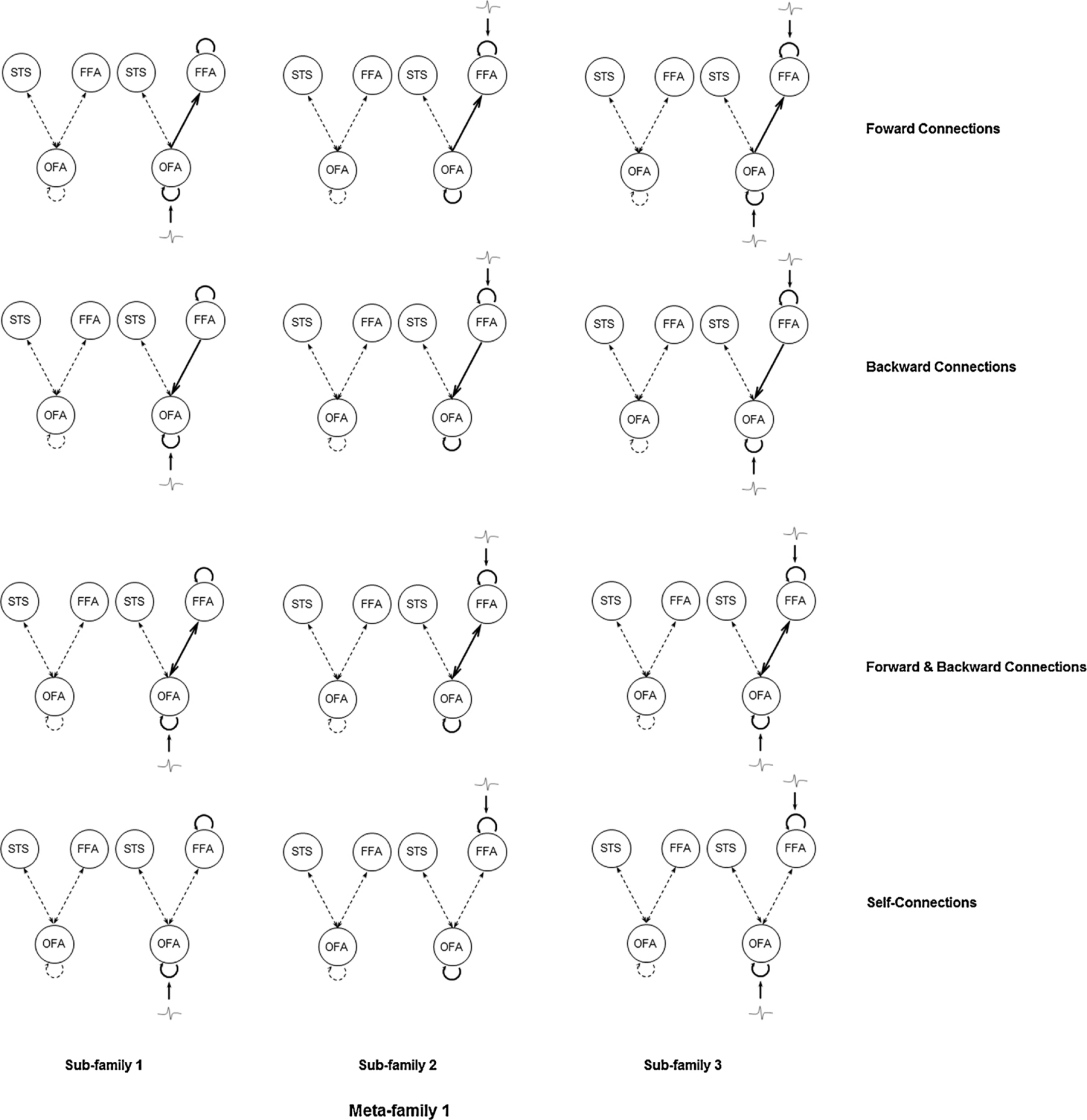
Meta-family 1. All models in Meta-family 1 have the simple model space with connections between OFA, FFA, and STS within each hemisphere. Sub-family 1, sub-family 2, and sub-family 3 have models with driving inputs entering OFA only, FFA only, and both OFA and FFA respectively in each column. Models within each sub-family differed from each other in terms of the type of modulations enabled by face repetitions for changes in forward and self-connections only, backward and self-connections only, both forward/backward and self-connections, or self-connections only. OFA, occipital face area (inferior occipital gyrus); FFA, fusiform face area (fusiform gyrus); STS, superior temporal sulcus.

**Fig. 4:**
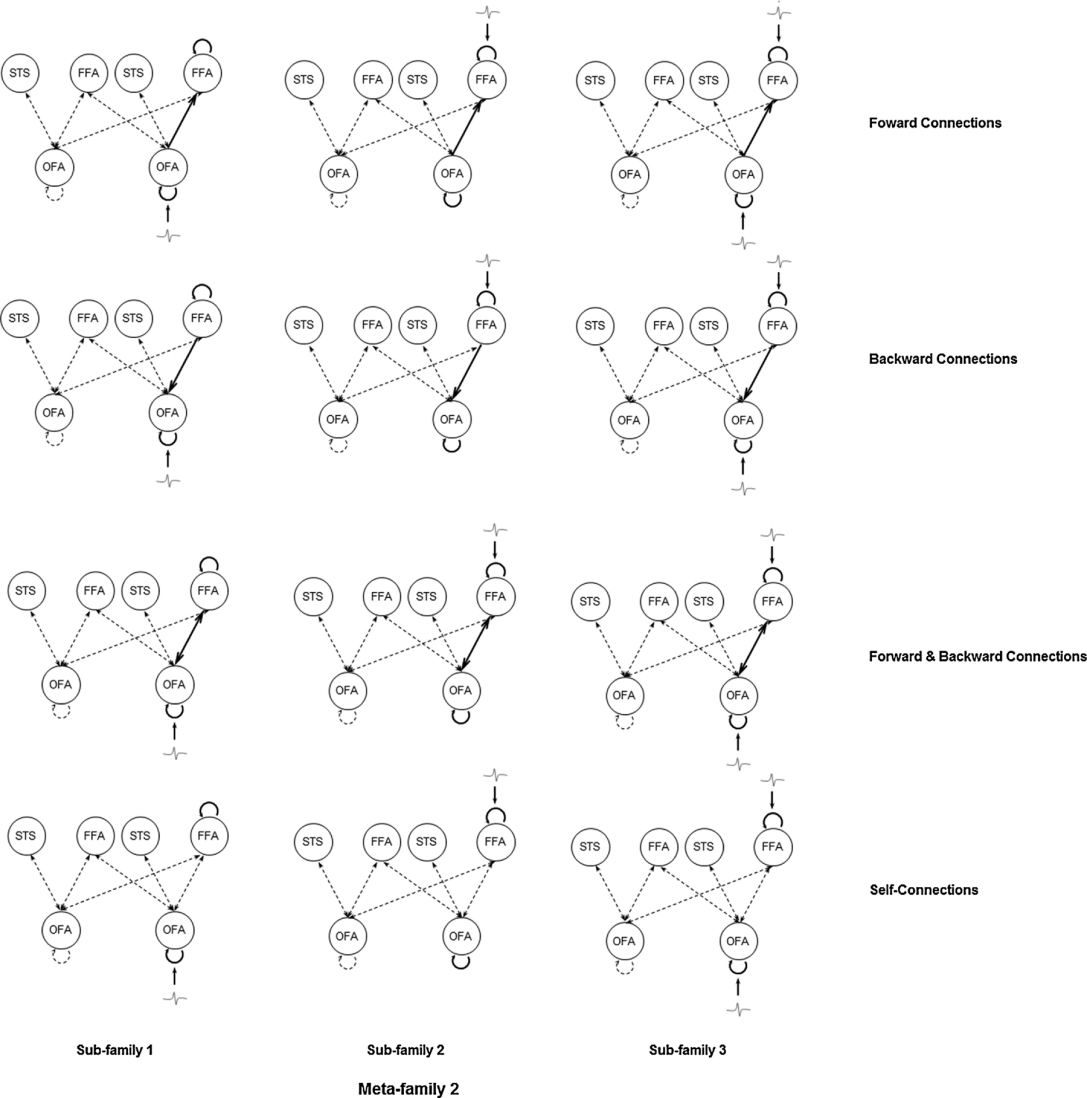
Meta-family 2. All models in Meta-family 2 have the inter-hemispheric model space with extra inter-hemispheric connections between OFA and FFA on top of the simple model space. Sub-family 1, sub-family 2, and sub-family 3 have models with driving inputs entering OFA only, FFA only, and OFA and FFA respectively in each column. Models within each sub-family differed from each other in terms of the type of modulations enabled by face repetitions for changes in forward and self-connections only, backward and self-connections only, both forward/backward and self-connections, or self-connections only. OFA, occipital face area (inferior occipital gyrus); FFA, fusiform face area (fusiform gyrus); STS, superior temporal sulcus.

We then specified a set of 24 models based on this two symmetric hemisphere model space with systematic variations in face repetition modulation between OFA and FFA in the right hemisphere. Similar connectivity modulations on recurrent connections between FFA and OFA during face repetition tasks have been tested in a DCM-fMRI study (Ewbank et al., 2012). The right hemispheric dominance of face detection and identification has been consistently reported over the last two decades of neuroimaging research in infants (e.g., de Heering and Rossion, 2015), children (e.g., Kuefner et al., 2010) and adults (e.g., Ewbank et al., 2012). Meta-family 1 (see Fig. 3) has models derived from the simple model; Meta-family 2 (see Fig. 4) includes connectivity variations of the inter-hemispheric model. Each Meta-family was then composed of three sub-families, with inputs modelled to enter the OFA only (sub-family 1), FFA only (sub-family 2), and both the OFA and FFA (sub-family 3). These three alternative specifications of inputs were set up to test a rather recent view of face perception proposing that other than from the OFA, FFA receives independent input from low-level visual areas (Kim et al., 2006) and is activated in parallel with OFA for the process of a coarse-to-fine representation generation of faces (Rossion and Caharel, 2011). Lastly, within each sub-family, there are four alternative models differed in whether face repetitions modulate the efficacy of the forward connections, the backward connections, both the forward and backward connections between OFA and FFA, or only self-connections of OFA and FFA.

All 24 models were inverted individually, and the random-effects Bayesian model selection (BMS-RFX) was used to compare the exceedance probability (i.e., the probability of each model more likely than any other tested models to have generated the observed data) of each individual model or model family to select the most possible model (the “winning model”) across individuals within each group (Stephan et al., 2010).

Model inversion was restricted within the time window of the M250, where the repetition effects in the ERFs were significant (adults: 0–212 ms; children: 0–326 ms, see below sensor space results). This was to optimise the investigation of spatiotemporal dynamics of face stimuli within the network as a result of network perturbations from the experimental modulation. It is important to define the time window for DCM model inversion for accurate source reconstruction (Litvak et al., 2011). Model evidence was drawn firstly at the Meta-family level (Meta-family 1 vs. 2) using the family Bayesian model selection (BMS) method (Penny et al., 2010). A second level family BMS on models from the most likely Meta-family was then carried out to assess the most plausible sub-family, followed by the final BMS to examine which single model would perform best in explaining the observed sensor level data.

## 3 Results

### 3.1 Sensor space results

**Fig. 5:**
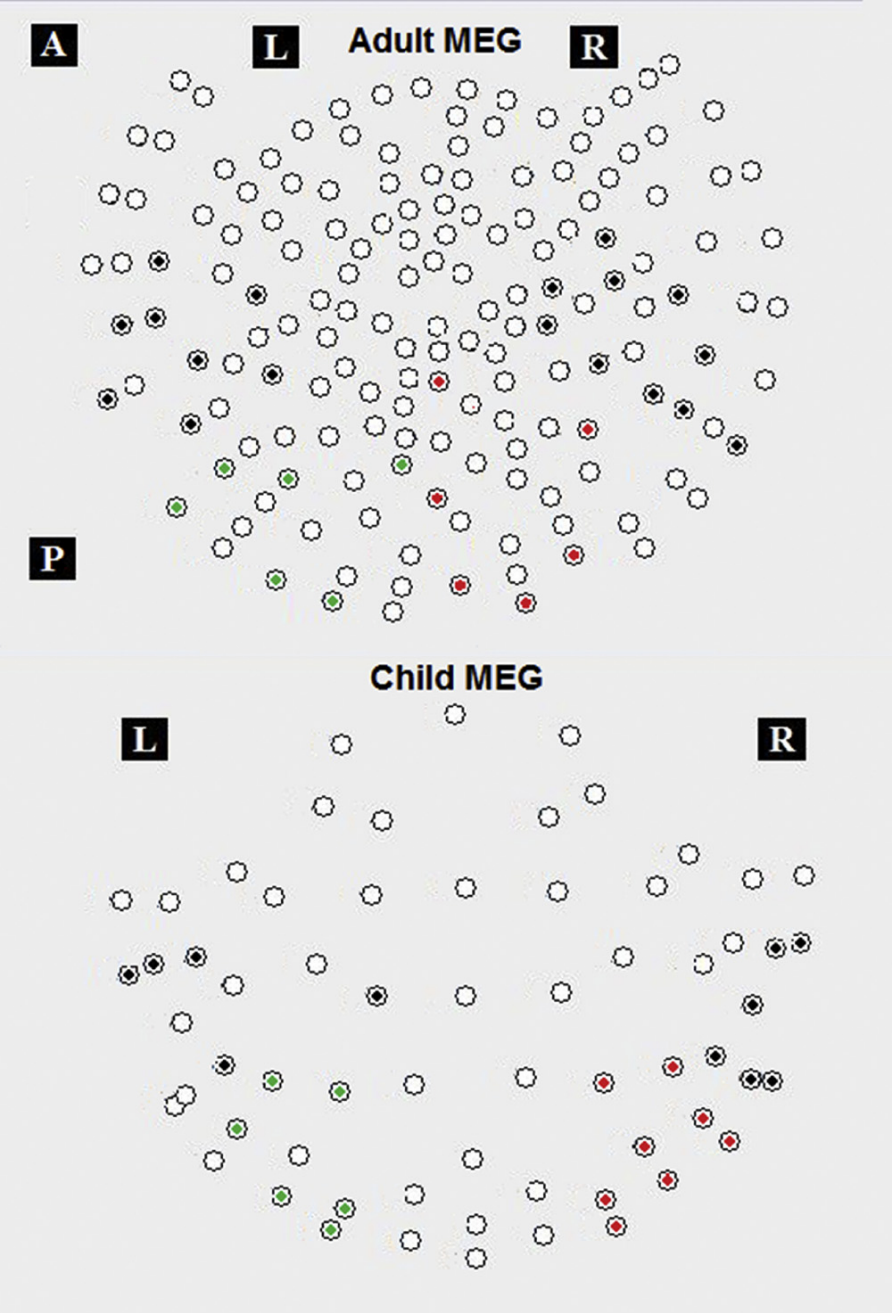
Sensors of Interest (SOIs) in adults (N = 11, top panel) and children (N = 10, bottom panel), from which significant face-evoked components (i.e., M100, M170 and M250) were identified showing a significantly larger amplitude to faces than baseline. Black dots for bilateral temporal sensors, red for right occipital and green for left occipital sensors.

**Fig. 6:**
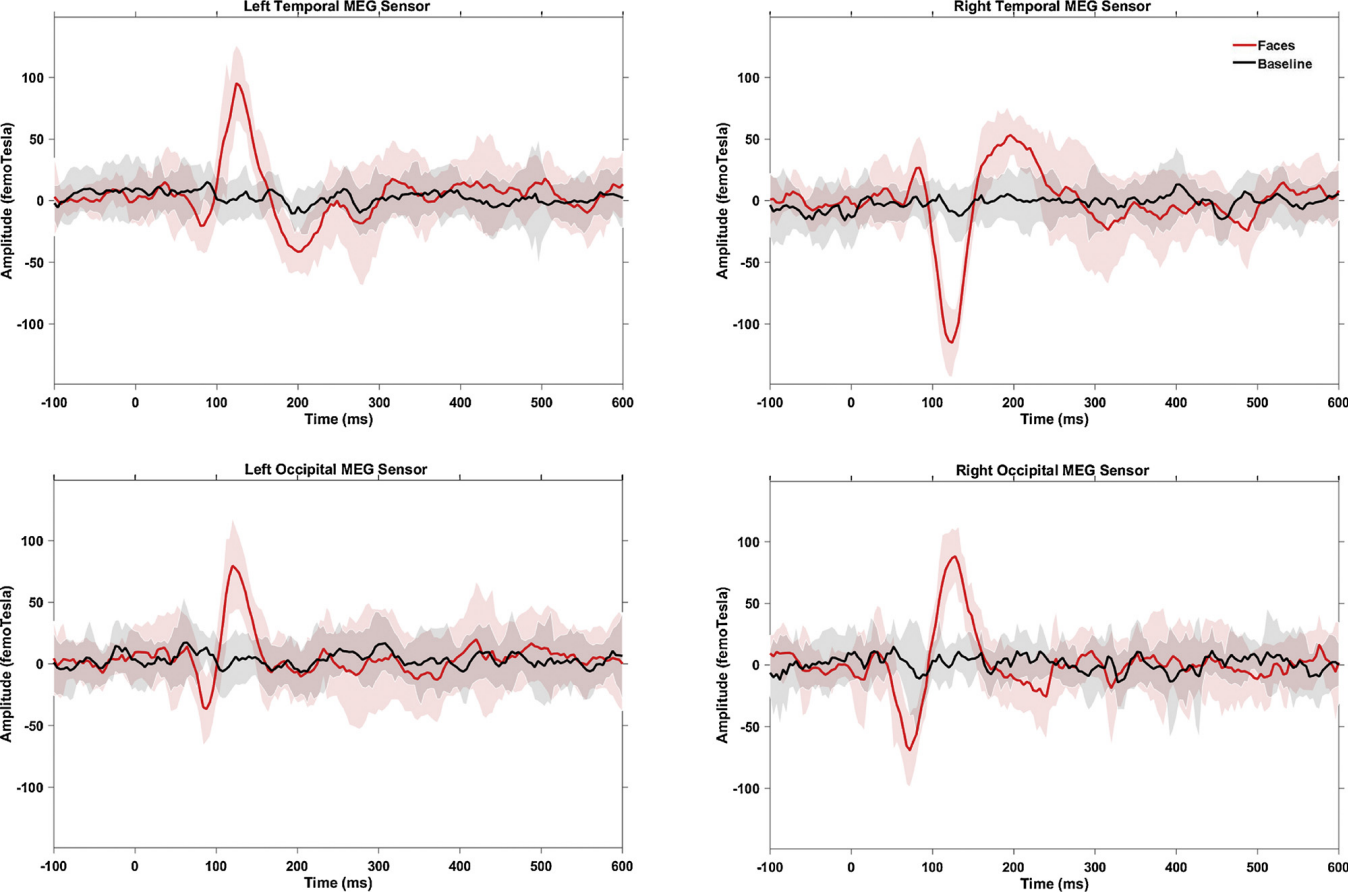
Averaged sensor waveform from 30 out of 160 MEG sensors in each hemisphere from adults (N = 11). Black line shows baseline responses and red line indicates responses to faces (novel plus repeated faces). Shaded areas represent the corresponding 95% confidence intervals across all participants in the group.

**Fig. 7:**
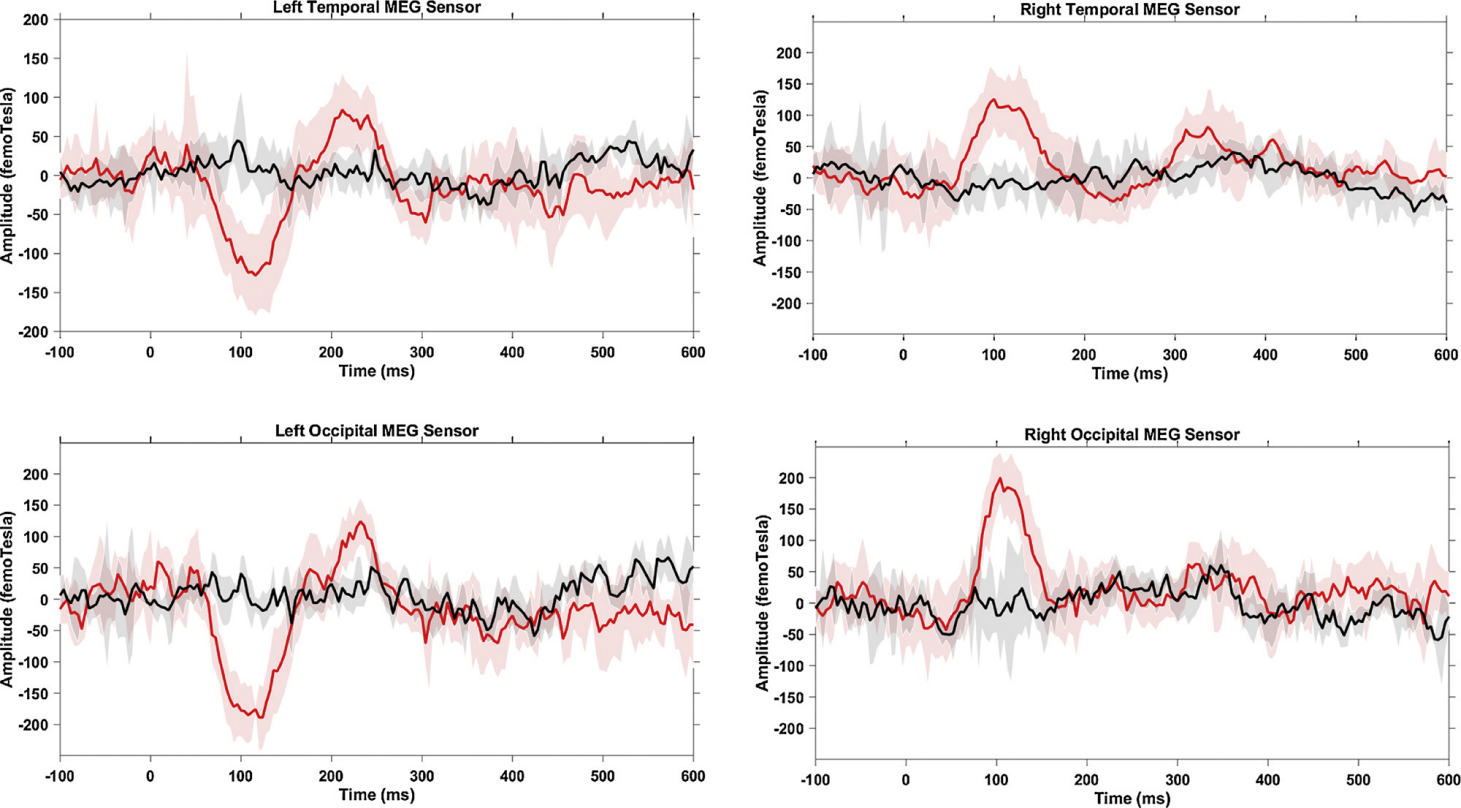
Averaged sensor waveform from 25 out of 64 MEG sensors in each hemisphere from children (N = 10). Black line shows baseline responses and red line indicates responses to faces (novel plus repeated faces). Shaded areas represent the corresponding 95% confidence intervals across all participants in the group.

A data driven topographical analysis was carried out, providing a statistic parametric map of spatial and temporal clusters of face-sensitive responses, rigorously correcting for family-wise error rates (FWE). Two bilateral occipitotemporal clusters of sensors were obtained (Fig. 5), giving significantly greater ERFs to faces than baseline (FWE-corrected *p <* 0.05) in adults (30 out of 160 channels) and children (25 out of 64 channels). Adult ERF waveforms were characterised by three face-evoked responses (Fig. 6): M100, latency around 85 ms (range: 63–110 ms); M170, around 137 ms (range: 115–183 ms); and M250, around 205 ms (range: 165–212 ms). ERFs of children (Fig. 7) showed a prominent M100 component peaking at 117 ms (range: 90–167 ms); an M170 response at 214 ms (range: 167–277 ms), and a M250 response at 305 ms (range: 260–326 ms).

**Fig. 8:**
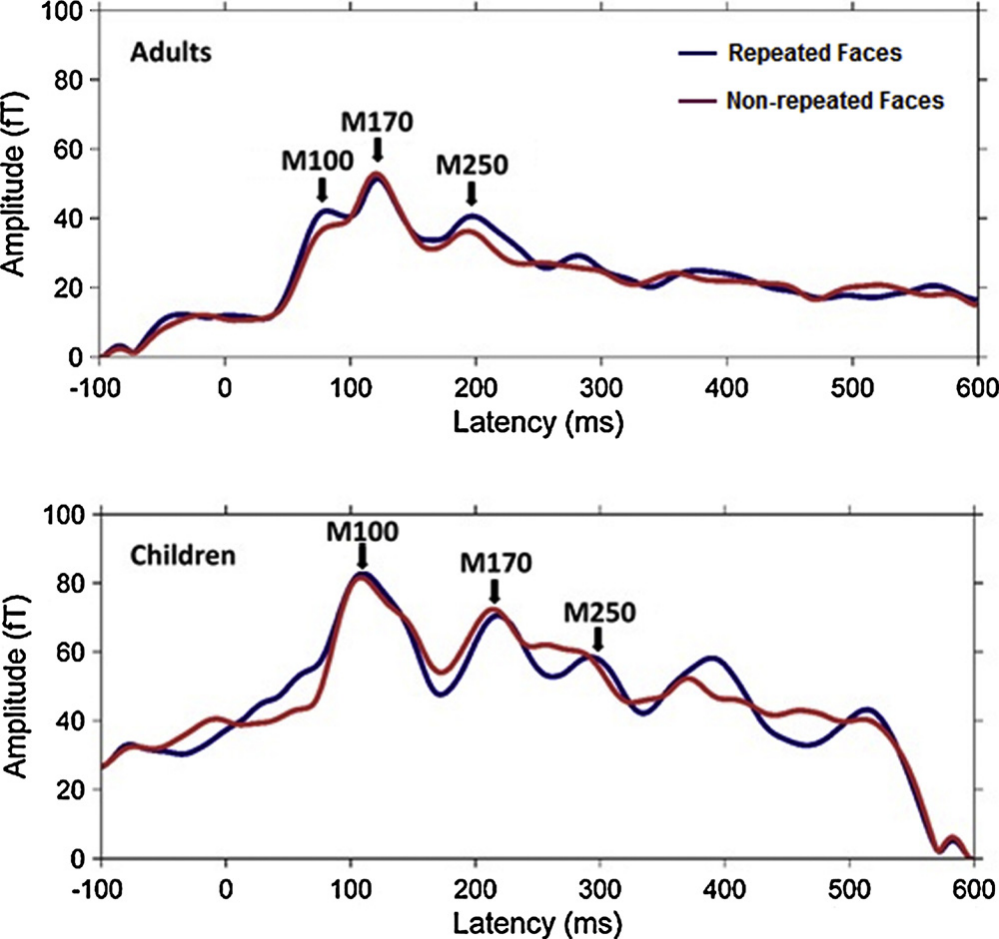
Root-mean-square waveforms for two experimental conditions from sensors of interest in adults (N = 11, top panel) and children (N = 10, bottom panel). Blue line shows responses to repeated faces and red line indicates responses to novel faces.

Fig. 8 shows root-mean-square (RMS) waveforms for each group. Mean latencies and amplitudes for three ERF components from SOIs were computed in each participant, and a paired-*t*-test with FDR correction was computed to examine experimental effects within each group. In adults, the only significant effect was a larger M250 (∼6.45 fT) for repeated faces compared to non-repeated ones (*t* (10) = 4.25, *p* = 0.002, *p*-fdr = 0.012); no significant effects was obtained for M100 or M170 responses. In children, no significant amplitude effects were found for any of the three components; however the M250 had an earlier latency (∼22.15 ms) for repeated than non-repeated faces (*t* (9) = 4.43, *p* = 0.002, *p*-fdr = 0.012).

### 3.2 DCM-bayesian model selection (BMS)

**Fig. 9:**
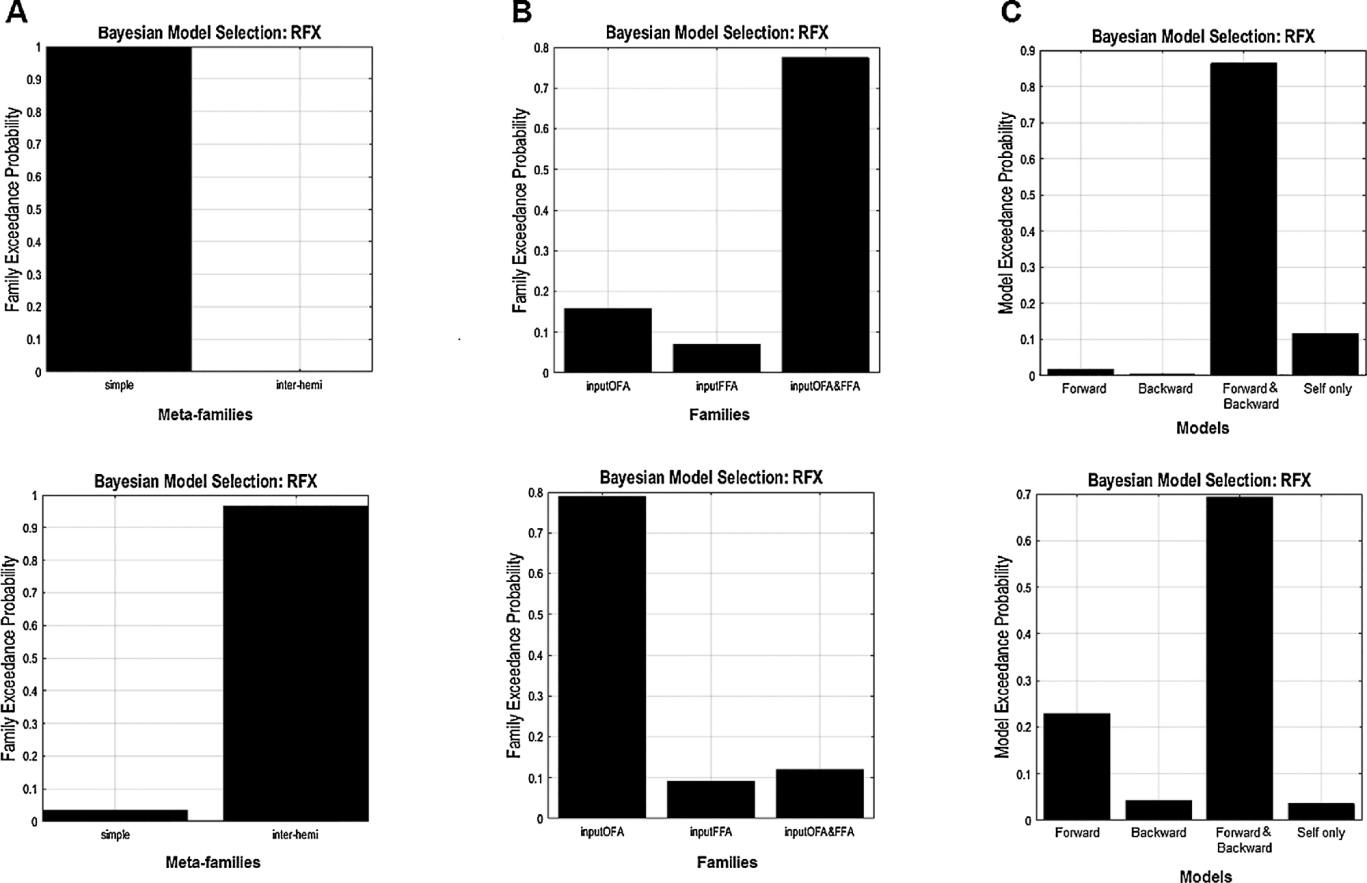
Bayesian model selection with random effects (BMS-RFX) (11 adults and 10 children). (A) Meta-family inference based upon the two basic model space: In adults (upper panel), the BMS favoured the simple model space − connections between OFA, FFA and STS within each hemisphere; in children (lower panel), BMS-RFX selected the inter-hemispheric model where extra inter-hemispheric connections between OFA and FFA are in place. (B) Sub-family inference based upon the location of the driving input entering the model space: In adults (upper panel), the second level BMS-RFX favoured sub-family 3, which has inputs entering both the OFA (occipital face area/inferior occipital gyrus) and the FFA (fusiform face area/middle fusiform gyrus); in children (lower panel), BMS-RFX selected Family 1, in which models have inputs entering the OFA only. (C) The final level BMS-RFX on four alternative models within the winning sub-family respectively, in both groups, clearly favoured the model with modulations on forward and backward connections between and self-connections within FFA and OFA in both adults (upper panel) and children (lower panel).

**Fig. 10:**
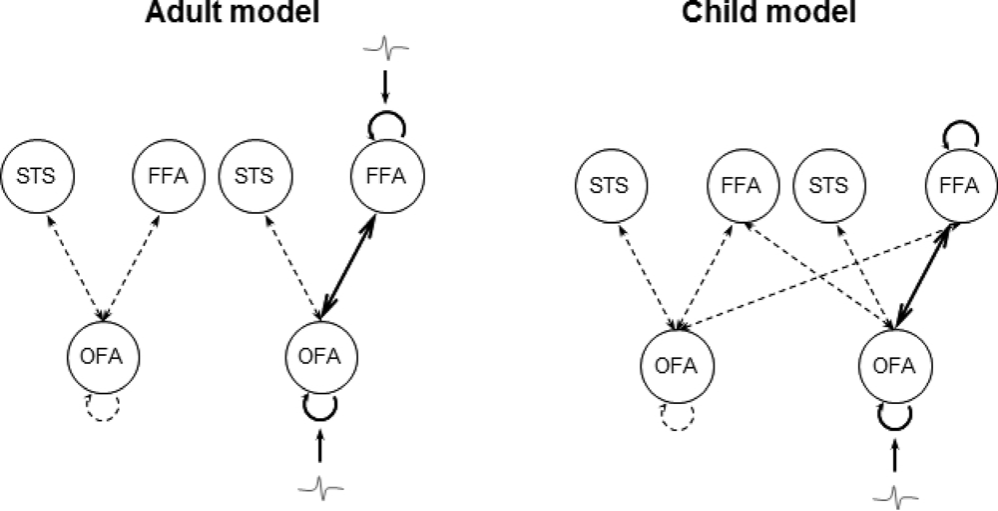
The winning model of adults (Left panel, N = 11) and children (Right panel, N = 10). Compared to the adult winning model, the child winning model has inter-hemispheric connections between OFA and FFA on top of the intra-hemispheric connections between OFA, FFA and STS. Bayesian model selection with random effects (BMS-RFX) preferred driving inputs enter into both OFA and FFA in adults and into OFA only in children. Both models have reciprocal connections between and self-connections within the OFA and FFA that are responsive to face repetition modulations. OFA, occipital face area (inferior occipital gyrus); FFA, fusiform face area (fusiform gyrus); STS, superior temporal sulcus.

In adults, DCM models were inverted within 0–212 ms (the time window of M250) for each individual. Consistent with our previous study (He et al., 2015), at the Meta-family level the simple model structure (Meta-family 1) was found to be superior in explaining face-selective MEG response in adults by the random-effects Bayesian model selection (BMS-RFX; exceedance probability = 0.99, Fig. 9A upper panel). The second level family BMS-RFX model selection showed that sub-family 3, with driving input entering both OFA and FFA, has the highest exceedance probabilities (0.78) (Fig. 9B upper panel). The final BMS-RFX among models with different types of modulatory connections favoured the model (in Fig. 10 left side) that has all connections dynamically change in response to the face repetition, including the forward and backward connections between the OFA and FFA, as well as the self-connections within the two regions (exceedance probability = 0.87, Fig. 9C upper panel).

In children, DCM models were also inverted for each individual within the time window of M250 (i.e., 0–326 ms). At the first Meta-family level of model selection, BMS-RFX clearly favoured Meta-family 2 (exceedance probability = 0.96, Fig. 9A lower panel). The second level family BMS-RFX showed strong preference to sub-family 1, which consists of models with inputs only entering OFA (exceedance probability = 0.79, Fig. 9B lower panel). In the final BMS-RFX, the model (in Fig. 10 right side) that has recurrent backward and forward connection between OFA and FFA, with changes in self-connectivity in both regions, was preferred in explaining the face repetition effect in children (exceedance probability = 0.68, Fig. 9B lower panel).

Across individuals in both groups, the three levels of BMS-RFX showed highly consistent results with a comparable variability between children and adults (see details in Fig. 3 in the Supplementary Materials).

## 4 Discussion

The results of the present study confirm that M250 amplitude is significantly modulated by face repetition in the adult brain (Schweinberger, 2011, Schweinberger et al., 2007, Simpson et al., 2015). In contrast, using a pediatric MEG system, children showed no significant M250 amplitude effect but did exhibit a modulation of M250 peak latency, with significantly faster responses to repeated faces than to non-repeated faces. This latency effect has been previously reported in EEG measurements of infants (Schweinberger et al., 2002). DCM analyses demonstrated that the key group difference in effective connectivity for this time window lies in the visual inputs entering into the reciprocal network of OFA and FFA: a single input to the OFA in children, but parallel inputs through both the OFA and FFA in adults.

Our MEG data showed three brain responses − the M100, M170, and M250 in adults at latencies consistent with previous reports (Schweinberger et al., 2004, Schweinberger et al., 2007, Schweinberger et al., 2002, Simpson et al., 2015, Taylor et al., 2011a). As expected, there were neither amplitude nor latency changes of the M100 and M170 for face repetitions, suggesting insensitivity of these components to this manipulation. This finding agrees well with the standard model of the temporal structure of face processing, which proposes that the M100 represents the general low-level perceptual analysis of visual stimuli (e.g., colour and phase-spectrum), and the M170 indexes the earliest categorical encoding of facial structural information (e.g., integration of facial features into a face representation) (Eimer, 2011, Rossion and Caharel, 2011, Simpson et al., 2015). Our finding of a repetition-sensitive M250 in adults is consistent with the recent consensus that the M/N250 indexes the computation of identity, or the formation of individual face exemplars, subsequent to the structural encoding of faces (Schweinberger et al., 2007, Schweinberger et al., 2002, Simpson et al., 2015, Taylor et al., 2010).

In children, M100, M170 and M250 signals over occipitotemporal areas manifested no amplitude sensitivity to face repetitions. However, children showed a faster M250 peak latency for repeated than non-repeated faces. This finding is consistent with previous reports of reduced N290 latency in 9-month-old infants (Peykarjou et al., 2016) and reduced N170 latency in adults (Itier et al., 2006) for repeated unfamiliar faces. To the best of our knowledge, there are no other published MEG/EEG studies that have investigated the face repetition effect in preschool children. There is a MEG study in school-age children (6–7 years) that reported an enhanced brain response to repeated faces, but with a much broader time window (280–680 ms) that was only identifiable at the source level (Itier et al., 2006, Taylor et al., 2011b). It is unclear whether the M250 component found in our preschool children is the functional precursor of the much broader component reported in those 6–7-year-olds. The absence of the M250 amplitude sensitivity to face repetitions may be due to the design of our particular paradigm. The processing effort upon the immediate repetition of unfamiliar faces is high in children, as reflected in the reduced M250 latency; however, more repetitions are required for building a stable representation. Therefore, either increasing the number of repetitions of the same face, or increasing the duration of each presentation, might help to elicit face repetition effects on the M250 amplitude in children.

Results from the DCM analysis reveal that both the adults and preschool children recruit reciprocal connections between OFA and FFA for computing identity-specific facial attributes. These results mirror the changes found in a recent fMRI connectivity study showing differential weighting of forward and backward OFA to FFA connections during processing of repeated faces (Ewbank et al., 2012). Strikingly, the patterns of effective connectivity within the OFA and FFA network were significantly different for the two age groups (Fig. 10) in terms of how the visual inputs enter the network. We tested three alternative models, i.e., inputs coming either through OFA only, FFA only, or both OFA and FFA. The Bayesian model selection in adults clearly favoured dual inputs of facial information to the network, i.e., inputs directly into the OFA and FFA (Fig. 7A). This finding is in line with reports from lesion studies in which the functionality of the right FFA and superior temporal sulcus (another core face region) were found to be intact in prosopagnosia patients, despite structural damage to the right OFA (Rossion et al., 2011, Schiltz and Rossion, 2006) or to bilateral OFAs (Steeves et al., 2006). Moreover, structural imaging studies have also reported direct connections from early visual areas to the FFA (Gschwind et al., 2012, Pyles et al., 2013). Therefore, our data adds evidence to the argument that in the mature human brain, both the OFA and FFA receive inputs through multiple pathways from the low order visual areas to compute face representations collaboratively and independently.

The main difference of the winning model for children compared to adults is the single input through OFA to the network. This pattern suggests that the fusiform gyrus in the immature brain has weaker connections with lower visual areas and, therefore, receives less information to operate on face-related processes for invariant identity specific features. This is consistent with recent fMRI evidence using DCM, showing weaker connections between lower visual areas and the fusiform gyrus in children compared to adults during performance of a face identification task (Fairhall and Ishai, 2006). It has also been shown in some recent connectivity studies that cortical networks for face processing are continuously reorganised and strengthened in such a way that some of the connections linked to child face-processing skills would be replaced by maturing connections emerging during the development of face recognition strategies (Johnson, 2001, Johnson, 2011, Joseph et al., 2011). If this is so, the lack of significant effective inputs to the FFA in children might result in a weaker guidance of the fine-tuning of processing in the OFA for the computation of facial identity attributes, manifest at the sensor level as a lack of M250 response to face repetitions.

A number of conceptional and methodological limitations apply to the current study. First, an interleaved design with repeated faces intervened by a varying number of different faces may be more optimal for minimising expectations about the nature of the next stimulus. Second, since we used DCM models derived from the previous literature, the present data cannot rule out the existence of comparable or better models. Future studies should endeavour to examine whether the current findings can be reproduced by modelling with more extended networks based on additional priors from advanced source reconstruction techniques or multimodal recordings (Friston et al., 2017). Our study is also limited by the relatively small number of participants in our two groups: larger sample sizes would be preferred to increase statistical power. Finally, as pointed out by one of our reviewers, it would also be useful in future pediatric studies to assess whether there are any important differences (e.g., personality) of children who are compliant during the scanning procedure compared to those that are not able to follow task instructions.

In conclusion, this is the first study of the effect of face repetitions on the M250 in preschool children. The MEG results show that immediate repetitions of faces enhance the amplitude of the M250 neuromagnetic component in adults, but had no influence on earlier components such as M100 and M170. However, in children, repetitions significantly modulated the latency but not the amplitude of the M250 peak; there were no significant amplitude or latency effects on earlier peaks. Most importantly, by using the DCM connectivity analysis, this study examined the functional connectivity between two key face-sensitive brain regions, the OFA and FFA. Results show that the generation of the M250 in both adults and children relies on reciprocal connections between the OFA and FFA. In adults this network receives inputs from lower order visual areas through both OFA and FFA, but in children only the OFA receives these inputs. Overall, this investigation of the functional profile of the M250 component following immediate face repetition in preschool-aged children provides valuable insights into our understanding of the spatiotemporal characteristics of the development of the face processing network. Moreover, it also represents an important advance in the application of the pediatric MEG to elucidate the developmental trajectories of transient cognitive process and its underling neural organisation in both space and time. Future applications of our pediatric MEG in studying the preschool cognition of face processing may benefit from engaging both the core and extended face networks with (1) more repetitions of different faces, (2) longer presentation for each face stimulus, (3) more demanding explicit recognition tasks, and (4) well-controlled comparison stimuli (e.g., objects) in order to address a comprehensive account of the development of face selectivity in human brain.
