# Supplementary material for: Development of face recognition: Dynamic causal modelling of MEG data
Source: Dev Cogn Neurosci. 2017 Nov 23;30:13–22. doi: 10.1016/j.dcn.2017.11.010 (PMC6969123; doi:10.1016/j.dcn.2017.11.010)
Supplement: Supplementary file 1 [file mmc1.docx]

**Supplementary Materials**

*Development of face recognition: Dynamic causal modelling of MEG data*

**1. Quantitative comparison of ERFs between 1^st^ and 4^th^ experimental block**

To further account for the potential expectation effect of highly repetitive stimulus at the group level, additional analyses of ERFs across the whole sensor array were performed using the statistical parametric mapping in SPM8, with ‘block’ as a within-subject factor in both child and adult groups. Results showed no significant effect on either repeated or non-repeated faces between the 1^st^ and 4^th^ (last) block (FWE corrected *P* > 0.05, see also Supplementary Figure 1 and Figure 2 for the root mean square waveforms from SOIs in children and adults). These results suggest no significant correspondent expectation differences to the repetition effects found in both groups over the course of trials.


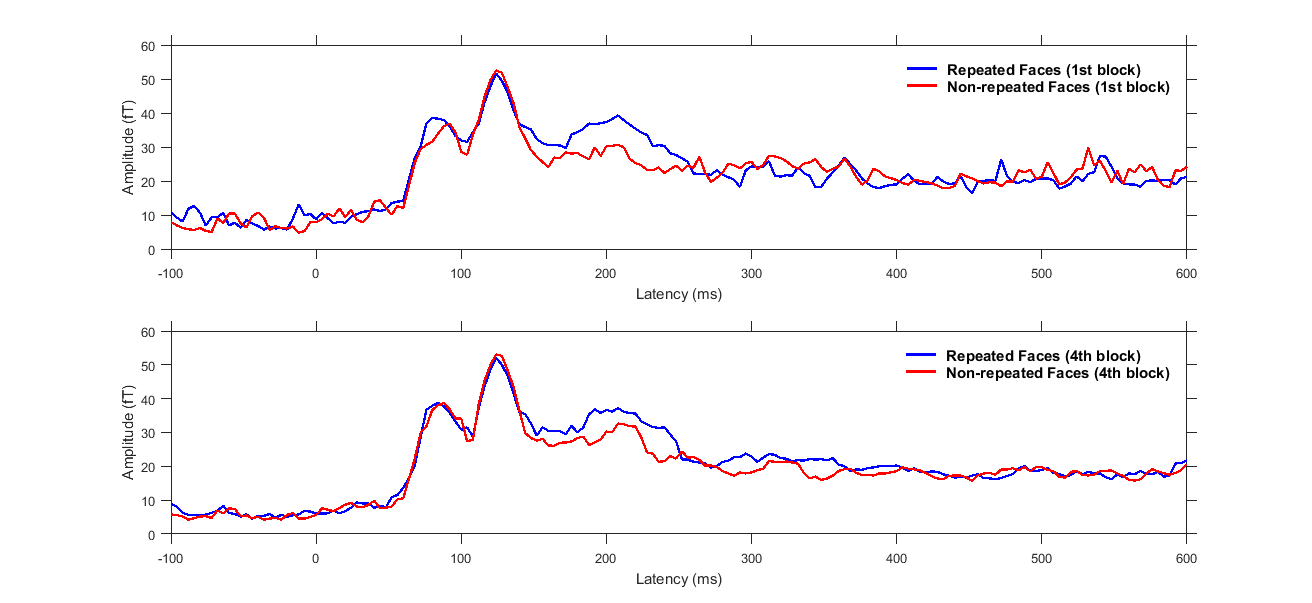


**Supplementary Figure 1.** Root-mean-square waveforms for two experimental conditions from sensors of interest in adults (N = 11) for the 1^st^ block (top panel) and the 4^th^ block (bottom panel). Blue line shows responses to repeated faces and red line indicates responses to novel faces.


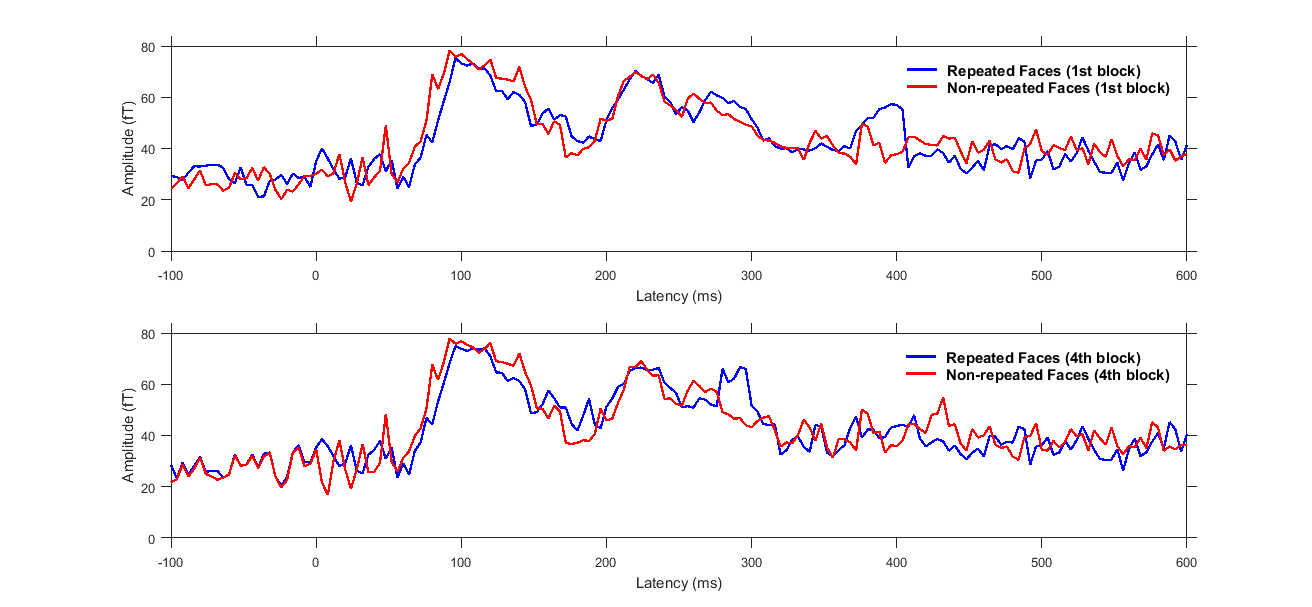


**Supplementary Figure 2.** Root-mean-square waveforms for two experimental conditions from sensors of interest in children (N = 10) for the 1^st^ block (top panel) and the 4^th^ block (bottom panel). Blue line shows responses to repeated faces and red line indicates responses to novel faces.

**2. Individual results of Bayesian model selection with random effects (BMS-RFX)**

The BMS-RFX results for each individual data re-illustrated in Supplementary Figure 3. Columns 2-3 demonstrate that both adults and children have good consistency in the model preferences at the Meta-family level (100% for the winning model in adults and 90% in children). Within each sub-family (columns 4-6), both groups show 100% consistency of individual model preferences among the three sub-families. Adults show slightly higher consistency of individual model preferences among four types of models (64% for the winning model), whereas children have slightly more variability in the model preference at this level (60% for the winning model).


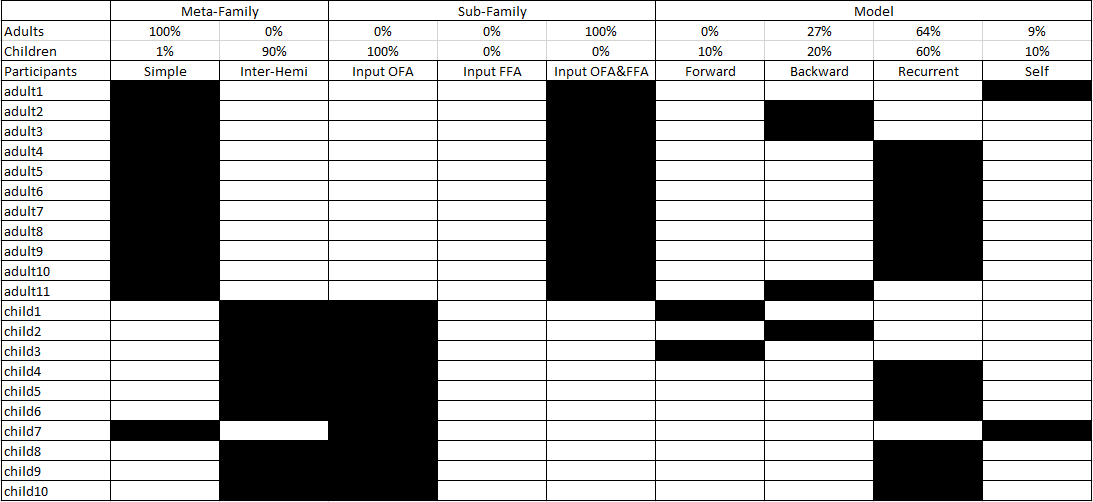


**Supplementary Figure 3.** Individual results of Bayesian model selection with random effects (BMS-RFX). Solid cells indicate the winning model families/models at the Meta-family level, sub-family level and individual model level.
